# Supplementary material for: Association between Social Engagements and Stigmatization of COVID-19 Infection among Community Population in Japan
Source: Int J Environ Res Public Health. 2022 Jul 25;19(15):9050. doi: 10.3390/ijerph19159050 (PMC9329772; doi:10.3390/ijerph19159050)
Supplement: Supplementary file 1 [file ijerph-19-09050-s001.zip › ijerph-1824637-supplementary.pdf]

Association between social engagements and stigmatization of COVID-19 infection among community population in Japan

Supplementary materials

Supplementary Figure S1. Distribution of stigma total score

Supplementary Table S1. Stigma questionnaire items and results of principal component analysis

Supplementary Table S2. Mean score of stigma total score by demographic factors

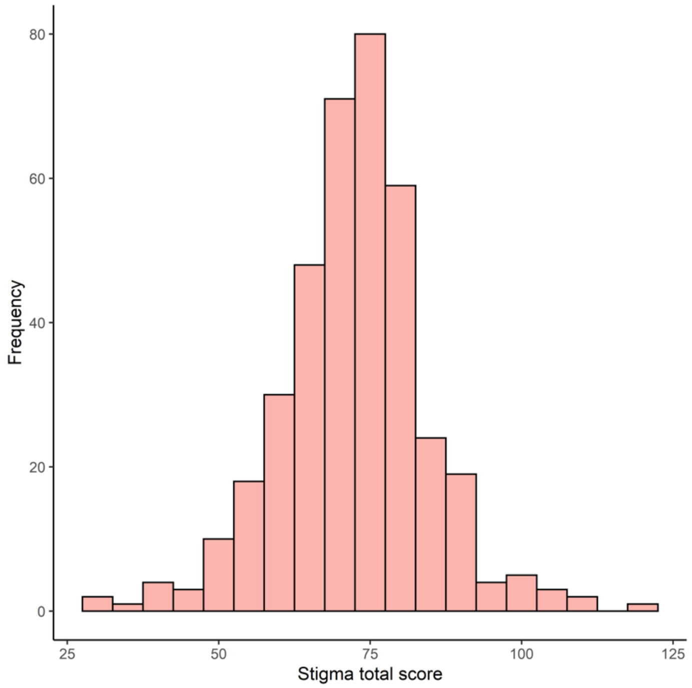

Supplementary Figure S1. Distribution of stigma total score

Supplementary Table S1. Stigma questionnaire items and results of principal component analysis

Items	Mean (SD)	Factor loadings		
		Social punishment	Self-deserve	Fear of infected
You were infected: Will lose my friends.	2.1 (0.93)	**0.73**	0.00	0.04
Someone infected: Have to move.	1.4 (0.62)	**0.63**	0.31	-0.26
Someone infected: Want to avoid being involved with them in the future.	1.5 (0.65)	**0.62**	0.17	-0.26
You were infected: Will lose my job.	2.0 (1.07)	**0.62**	0.00	0.17
Someone infected: Could not be helped if losing their job.	1.7 (0.83)	**0.61**	0.47	-0.12
Someone infected: They must hide the infection route.	2.0 (0.80)	**0.60**	0.29	0.06
You were infected: Have to move.	1.7 (0.83)	**0.60**	0.05	0.01
Someone infected: Annoying.	2.1 (0.83)	**0.59**	0.26	0.13
You were infected: Will be avoided by my acquaintance.	2.8 (0.92)	**0.55**	-0.09	0.42
You were infected: Will die.	2.3 (0.98)	**0.47**	-0.17	0.26
You were infected: Want to hide the fact that I am infected.	2.3 (1.04)	**0.44**	-0.01	0.24
You were infected: Embarrassing.	2.3 (0.95)	**0.42**	0.25	0.26
You were infected: Bothersome to be hospitalized or medicated.	2.2 (1.05)	**0.35**	0.10	0.08
Someone infected: Serves someone right.	2.3 (0.88)	0.18	**0.81**	0.13
Someone infected: Their responsibility.	2.5 (0.91)	0.22	**0.79**	0.18
You were infected: My responsibility.	2.6 (0.94)	0.04	**0.77**	0.15
You were infected: Serves me right.	2.2 (0.96)	0.00	**0.76**	0.11
Someone infected: Could not be helped if criticized by others.	2.1 (0.86)	0.48	**0.62**	0.04

You were infected: glad that I got an antibody.	2.3 (0.92)	0.03	**0.31**	0.06
You were infected: May infect others.	3.6 (0.74)	0.13	0.06	**0.73**
You were infected: Will cause trouble for my family and workplace.	3.5 (0.73)	0.20	0.08	**0.68**
Someone infected: Want to avoid being involved with them for a while.	3.0 (0.81)	0.32	0.15	**0.51**
Someone infected: Risk of getting infected will increase.	2.8 (0.79)	0.38	0.17	**0.47**
Someone infected: Should be isolated.	3.2 (0.96)	0.25	0.07	**0.44**
You were infected: Will be criticized by others.	2.9 (0.87)	0.26	0.30	**0.43**
Someone infected: Will refrain from going out for a while.	2.8 (0.90)	0.35	0.06	**0.42**
Someone infected: Could not be helped.	2.9 (0.85)	-0.15	0.14	**0.41**
You were infected: Could not be helped.	2.8 (0.93)	-0.16	0.32	**0.35**
Someone infected: Want to do something to help.	2.7 (0.84)	-0.14	0.06	**0.31**
Someone infected: Nothing to do with me.	1.9 (0.74)	0.16	0.18	**-0.21**

Bold signified the main items loading to each component.

Supplementary Table S2. Mean score of stigma total score by demographic factors

		Mean (SD)	P-values [a]
Age			0.768
	18-65 years old	72.4 (11.2)	
	> 65 years old	71.9 (14.4)	
Sex			0.046
	Male	70.9 (12.5)	
	Female	73.4 (11.8)	
Educational attainment			0.028
	Junior/ high	72.3 (14.0)	
	Vocational	74.9 (11.3)	
	University/ graduate	70.4 (10.2)	
Household income (JPY)			0.124
	0 - < 3M	70.1 (14.0)	
	3 - < 6M	73.9 (11.7)	
	6 - < 10M	73.5 (11.6)	
	+10M	72.5 (8.33)	
Number of past diseases			0.675
	None	73.0 (12.6)	
	1	72.1 (11.6)	
	+2	71.6 (12.5)	
Depressive symptoms (K6 total score)			0.003
	0 - 4	70.9 (11.6)	
	5 - 12	75.7 (13.0)	
	+13	73.0 (13.0)	

a: P-values were calculated with t-test for age and sex, and with ANOVA for educational attainment, household income, the number of past diseases, and depressive symptoms.
